# How Surface and Substrate Chemistry Affect Slide Electrification

**DOI:** 10.1021/jacs.4c01015

**Published:** 2024-04-02

**Authors:** Benjamin Leibauer, Ognen Pop-Georgievski, Mariana D. Sosa, Yun Dong, Wolfgang Tremel, Hans-Jürgen Butt, Werner Steffen

**Affiliations:** † Max Planck Institute for Polymer Research, Ackermannweg 10, 55128 Mainz, Germany; ‡ Institute of Macromolecular Chemistry, Heyrovského nám. 2, 162 00 Prague, Czech Republic; § Chemistry Department, Johannes-Gutenberg University, Duesbergweg 10-14, 55128 Mainz, Germany

## Abstract

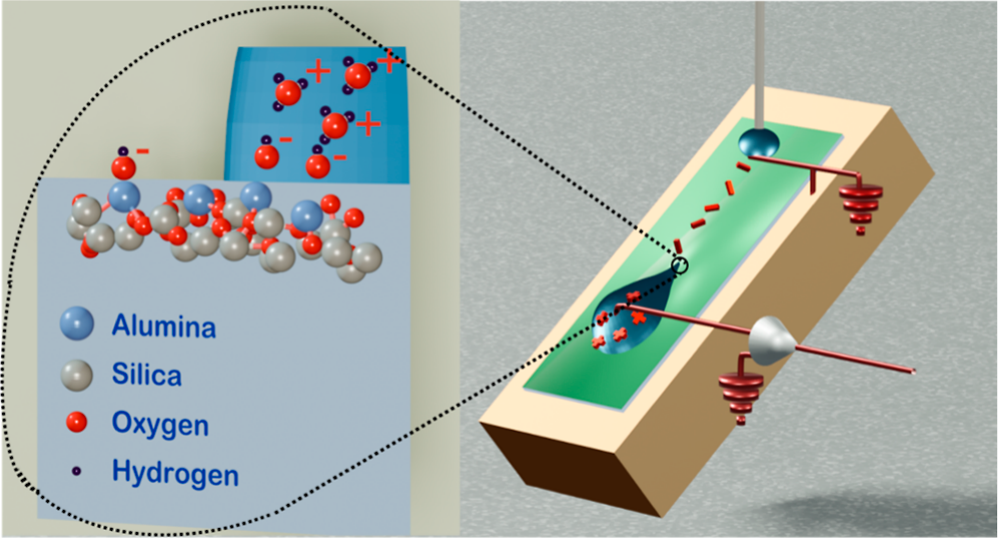

When water droplets move over a hydrophobic surface, they and the surface become oppositely charged by what is known as slide electrification. This effect can be used to generate electricity, but the physical and especially the chemical processes that cause droplet charging are still poorly understood. The most likely process is that at the base of the droplet, an electric double layer forms, and the interfacial charge remains on the surface behind the three-phase contact line. Here, we investigate the influence of the chemistry of surface (coating) and bulk (substrate) on the slide electrification. We measured the charge of a series of droplets sliding over hydrophobically coated (1–5 nm thickness) glass substrates. Within a series, the charge of the droplet decreases with the increasing droplet number and reaches a constant value after about 50 droplets (saturated state). We show that the charge of the first droplet depends on both coating and substrate chemistry. For a fully fluorinated or fully hydrogenated monolayer on glass, the influence of the substrate on the charge of the first droplet is negligible. In the saturated state, the chemistry of the substrate dominates. Charge separation can be considered as an acid base reaction between the ions of water and the surface. By exploiting the acidity (Pearson hardness) of elements such as aluminum, magnesium, or sodium, a positive saturated charge can be obtained by the counter charge remaining on the surface. With this knowledge, the droplet charge can be manipulated by the chemistry of the substrate.

## Introduction

The formation of charges at solid–gas interfaces after contact with liquids (i.e., charge separation^1^) is an universal problem, but it is still not well understood.^2^ There are several channels through which water droplets can spontaneously acquire a charge. At the end of the 19th century, raindrops were shown to be either positively or negatively charged.^3^ Further experiments demonstrated that a single water droplet becomes charged when it splashes onto a solid or liquid surface or flows through a tube.^1,4−6^ The sign of the charge can vary and be affected, for example, by the application of an electric field or the material of the tube.^1,5,6^ On this basis, it was found that a water droplet sliding over a polytetrafluoroethylene (PTFE) surface becomes charged. This effect became known as slide electrification.^7,8^ It is even possible to generate electrical energy from individual water droplets^9−12^ to power LEDs.^13^ Recently, it was found that the charging due to slide electrification can lead to electric potentials of up to 1 kilovolt.^14^

Slide electrification has been observed on many hydrophobic surfaces such as PTFE,^7,10,11,13^ fluorinated ethylene propylene,^9,15,16^ and fluorinated glass.^8,17,18^ In all these cases, the surface behind a sliding drop of water with a pH of around 5.5 (pH of DI water in equilibrium with air) becomes negatively charged.^19,20^ The droplet is positively charged by the remaining countercharges. The most likely process is that an electric double layer forms at the base of the droplet and that the interfacial charge remains on the surface.^2,11,17,20,21^ In the case of water, it is assumed that hydroxide anions (HO^–^) are formed by the autoprotolysis of water (2H_2_O ↔ H_3_O^+^+HO^–^) or are the product of an acid base reaction between the hydrophobic surface and the water.^11,15,19,21,22^ It is reported that the charge separation happens at the back of the droplet at the three-phase contactline.^23^ For some type of surfaces, the charging effects were explained in terms of an electron transfer between substrate and water droplet and models have been proposed.^24^

For slide electrification, only surfaces with receding contact angles (RCA) > 50° have been used. One reason is that droplets with smaller RCA no longer slide as easily. In addition, high RCAs appear to lead to higher droplet charges. One recently proposed charging mechanism attributes charge separation to the upward convective flow at the rear of the contact line. It leads to an increase in the screening length and, as long as the convection is stronger than the diffusion of counterions, the separation takes place at the rear contact line.^23^ It has been shown that the adsorption of protons or hydroxide ions can be influenced by surface modification. Surface-bound amine groups can be protonated as Brønsted bases so that the solid surface becomes positively charged and the droplet negatively charged (surface-NH_2_ + H_2_O → surface-NH_3_^+^ + HO^–^).^17^

Recently, the acid–base model (proton donor–acceptor) was used to describe the charge separation in slide electrification, in which a PTFE surface interacts as a proton acceptor or donor.^11^ In this context, the effect of pH^10,11,25^ and various dissolved ions^11,15,21,25^ on the droplet charge has been investigated. It has been proposed that the surface charge acquired by the sliding droplet and the ζ potential in the PTFE–water interface have the same physical basis. Therefore, under certain conditions, the same model can capture both charge in sliding droplets and ζ potentials.^11^

In contrast to PTFE, fluorinated glass has different substrate (bulk) and surface (coating) chemistry. For fluorinated glass, we have a hydrophobic surface bonded to a hydrophilic substrate. This system allows us to vary the substrate and surface chemistry.

**Figure 1:**
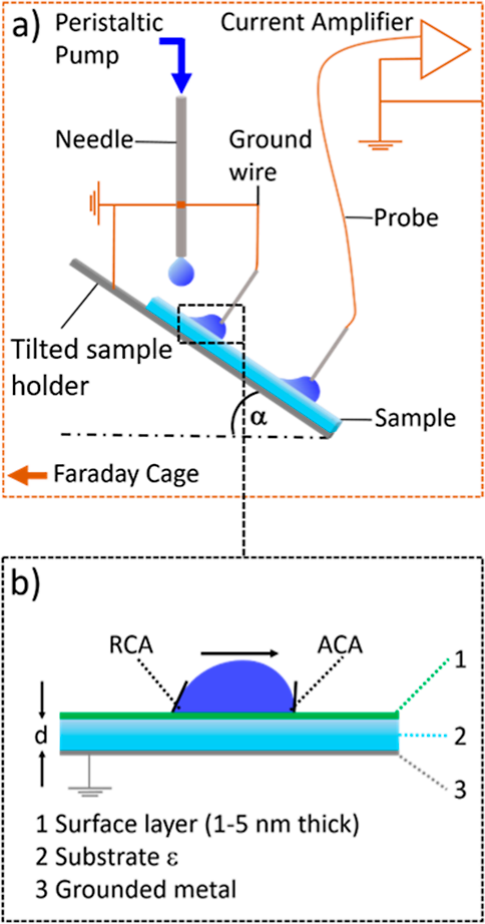
(a) Schematic of the experimental set up. (b) Relation between droplet, coating, and substrate. The thickness of the coating varies among the individual substrates. ε is the dielectric permittivity of the substrate and its thickness. ACA is the advancing contact angle and RCA the receding contact angle of the droplet.

In this work, we use different glass substrates and coatings to investigate the influence of surface and substrate chemistry on slide electrification. In general, glass consists of a network builder such as silicon dioxide (SiO_2_). By adding other metal oxides to the melt, other cations can be introduced into the network, thereby changing the physical (e.g., the dielectric permittivity ε_r_) and chemical properties of the glass.^26,27^ We demonstrated on flat hydrophobic surfaces that the dielectric properties influence the droplet motion by its dielectric permittivity ε_r_ and thickness *d* (Figure 1). The permittivity ε_r_ indicates how easily a material can be polarized in an electric field. The higher the dielectric permittivity, the better the surface charges are screened. The field strength of the deposited charge decreases and drop motion is less influenced by slide electrification.^20^ Since we measured a droplet charge in the order of nano-Coulomb and assumed that the droplet charge is generated by the adsorption of ions, we used the polarizability α to investigate the influence of the substrate chemistry.^8,11,17^ The polarizability can be determined by the Clausius–Mossotti relation. This equation links the permittivity ε_r_, a macroscopically measurable quantity, with the electric polarizability α a microscopic (molecular) quantity·^28^

In this work, we show that the substrate and the coating can change slide electrification in a more complex way. The glass was coated by chemical vapor deposition (CVD) with perfluorooctyltrichlorosilane (PFOTS)^8,17,20,29^ or octyltrichlorosilane (OTS)^30,31^ to generate a hydrophobic layer. To analyze the influence of both the substrate chemistry and the coating, we measured droplet charging for different substrates and different coatings. In addition, we tested the influence of the surface roughness. Our results can be used in future applications to increase the droplet charge.

## Experimental Section

### Substrates and Coating

Aluminosilicate glass (25 × 76 × 0.330 mm^3^, Schott AG, Germany), quartz glass slides (25 × 76 × 1 mm^3^, fused silica, Thermo Fisher Scientific, USA), sodium silicate glass (26 × 76 × 1 mm^3^, Thermo Fisher Scientific Gerhard Menzel B.V. & Co. KG, Germany), lanthanum silicate glass slides (25 × 76 × 1.5 mm^3^, Schott AG, Germany), and polytetrafluoroethylene foil (PTFE, thickness 0.05 mm, REIFF Technische Produkte GmbH, Germany) were used as substrates. The main difference in the chemistry of the glasses is the amount and type of their ionic components. Quartz glass contains only 0.01% of ionic components, sodium silicate glass 27.1%, aluminosilicate glass 40%, and lanthanum glass 75.1% (for details, see Supporting Information, Tables S1–S4).

Before applying the surface coating, the glass slides were rinsed with ethanol, cleaned with 2-propanol (both absolute, Honeywell, Germany) in an ultrasonic bath for 30 min, and then treated with hydrochloric acid (37%, Sigma-Aldrich, Germany) in methanol (VWR chemicals, France) (ratio 1:1) for 30 min^32^ Since a change in the chemical surface composition and the surface roughness (etching) of the lanthanum silicate glass was observed in the last step, no treatment with hydrochloride acid was carried out for the lanthanum silicate glass (Figure S1, Tables S5 and S6). After activation, the glass slides were rinsed three times with methanol and then with deionized and filtered water (DI water, 18 MΩ cm, obtained from an Arium Pro, Sartorius, Germany) and then dried with nitrogen gas. Subsequently, the surface was treated with an oxygen plasma (Diener electronic Femto, Plasma-Surface-Technology, Germany, 6 cm^3^ min^–1^ oxygen flow rate, 300 W) for 5 min. The glass slides were transferred directly to a desiccator. For CVD, 1 mL of 1H, 1H, 2H, 2H-perfluorooctyl-trichlorosilane (PFOTS, 97% Alfa Aesar) or octyltrichlorosilane (OTS, 97% Sigma-Aldrich, Germany) was placed in a glass Petri dish in the middle of the desiccator. The CVD reaction was carried out at a pressure of less than 200 mbar for 1 h at room temperature. After the reaction, the samples were rinsed three times with ethanol and DI water (to remove side products, see Figure S2), dried with nitrogen gas, and subsequently dried in a desiccator at less than 100 mbar for at least 2 h.^33^ Throughout the paper, we use the nomenclature for samples Coating@substrate.

### Atomic Force Microscopy

Surfaces were imaged in tapping mode (JPK NanoWizard 4, Bruker Nano GmbH, USA) with a cantilever (type OPUS, 160-AC-NA, back side coating with reflective aluminum) with 300 kHz resonance frequency, a spring constant of 26 Nm^–1^ and a nominal tip radius <7 nm (Figure S3). The calculation of the root mean square roughness (RMS) roughness was done with the software Gwyddion on part of the images of 15 × 15 μm^2^. The images were leveled using a polynomial background of first degree (offset and plane). Each process was repeated with three independent samples. Each sample was measured in at least two different positions. The standard error was calculated using the formula SE = σ × n^–1/2^, where σ represents the standard deviation and n represents the sample size. The measured root-mean-square roughness of the surfaces is summarized in Table 1.

**Table 1:** Measured Surface Roughness [RMS Over 15 × 15 μm^2^], Static Advancing (ACA) and Receding (RCA) Contact Angles and the Difference Between ACA and RCA (Hysteresis), Thickness of the Coating, the ζ Potential of the Coated Substrates ζ_c&s_, and Calculated ζ Potential of the Coating (ζ_c_). The ζ Potential Value Corresponds to the Measured ζ Potential at pH of 5.5

sample	RMS/nm	ACA/°	RCA/°	hysteresis/°	thickness coating/nm	ζ_c&S_/mV	ζ_c_/mV
PFOTS@sodium silicate glass	0.5 ± 0.1	119 ± 1	101 ± 3	18 ± 3	4.9 ± 1.8	–14 ± 2	12 ± 3
PFOTS@sodium silicate glass (sandblasted/1.5 bar)	9 ± 6	112 ± 2	80 ± 3	32 ± 2			
PFOTS@sodium silicate glass (sandblasted/2 bar)	104 ± 39	129 ± 1	98 ± 2	31 ± 2			
PFOTS@quartz glass	0.5 ± 0.1	116 ± 2	83 ± 2	33 ± 3	1.3 ± 0.1	–16 ± 2	1 ± 3
PFOTS@lanthanum silicate glass	0.5 ± 0.1	106 ± 1	80 ± 1	26 ± 1	5.6 ± 0.5	–23 ± 2	–6 ± 3
PFOTS@aluminosilicate glass	0.8 ± 0.1	111 ± 1	88 ± 2	23 ± 2	1.0 ± 0.1	–18 ± 2	9 ± 3
OTS@sodium silicate glass	0.7 ± 0.1	111 ± 2	97 ± 2	14 ± 3	1.5 ± 0.3	–4 ± 2	22 ± 3
OTS@quartz glass	0.5 ± 0.1	109 ± 1	89 ± 1	20 ± 1	1.3 ± 0.7	–20 ± 2	–4 ± 3
OTS@lanthanum silicate glass	0.5 ± 0.1	106 ± 2	90 ± 2	16 ± 3	0.8 ± 0.1	–13 ± 2	4 ± 3
OTS@aluminosilicate glass	1.6 ± 0.1	101 ± 1	89 ± 3	12 ± 3	1.1 ± 0.4	–19 ± 2	8 ± 3
PTFE foil (*d* = 0.05 mm)	116 ± 9	109 ± 2	88 ± 1	21 ± 2			

### Contact Angle Measurements

To characterize the wetting properties of the surfaces, ACA and RCA were measured with a goniometer (OCA35, DataPhysics Instrument GmbH, Germany). A DI water drop (6 μL) was placed with a Hamilton syringe (needle: blunt end, coated with PTFE, 51 mm length, 0.4 mm inner diameter) onto the horizontal surface. Side view videos were recorded and analyzed with the help of software SCA20 provided by DataPhysics Instrument GmbH. An LED (3000 K) was used as the light source. After placing a drop, its volume was increased and decreased by 40 or 50 μL (dependent on the surface) with a rate of 0.5 μL s^–1^ and 2 repeats. The measured ACAs, RCAs, and calculated contact angle hysteresis are summarized in Table 1. Errors were calculated by using standard deviation; each experiment was done with three different samples and three measurements at three different surface areas. The error in the contact angle hysteresis was calculated by Gaussian error propagation. We point out that the ACA and RCA for sliding drops can be different because they change with velocity, the degree of charge deposition, and the electric potential of the droplet. Only at the onset of sliding should both should match.

### Drop Charge Experiments

A custom-made experimental setup described previously was used (Figure 1a).^8^ The whole setup was placed in a Faraday cage. All metallic components were grounded (same ground). The sample was placed in a sample holder, and the tilt angle was adjusted to 50°. With a peristaltic pump (Gilson, MINIPULS 3, Wisconsin, USA), a series of water droplets were placed on top of the surface at a rate of 30 drops per minute. The needle used had an inner diameter of 2 mm, resulting in a drop size of 45 ± 2 μL. The needle was positioned 0.5 cm above the sample surface. To discharge possible charges in the droplet, the droplet first slides through a ground wire (Figure 1a). The distance between the ground wire and the electrode was 4 cm (sliding distance). A probe, connected to a current amplifier (DDPCA-300, FEMTO, Germany, gain of 10^6^ V/A with a raise time of 0.8 ms), was used to measure the discharge current of the droplets. By integration of the current signal over the discharging time, we calculated the drop charge of each droplet (Figure S4). The data were recorded using a multifunction box (National instruments, NI USB-6366, Hungary) connected to a computer. To electrically neutralize the surface of the sample prior to every drop charge measurement, an ion air blower (Simco-Ion, Aerostat PC Ionizing Air Blower, USA) was applied for 5 min. For the evaluation, we used the drop charge of the first droplet and the saturated drop charge. For this, we measured the charge of 500 droplets. The drop charge decreases with the increasing drop number. From the point where the drop charge reached a constant value, we averaged it to obtain the saturated drop charge (Figure S4). We measured three different samples three times at three different places. For the evaluation, we calculated the average value from these nine drop charges with the corresponding drop number of each sample. The standard deviation was then used to determine the measurement uncertainty of the droplet charge for each drop number, with the measurement uncertainty of the dropl charge at dropl number 250 being used for the measurement uncertainty in the saturated state (Figure S5). The averaged drop charges of all samples are compiled in Figure S6. We used fresh DI water for each experiment. At the time of use, the DI water had a pH of 5 5 due to the ubiquitous uptake of CO_2_ from the surrounding air.

### Spectroscopic Ellipsometry

Measurements were performed on a J.A. Woollam M-2000X spectroscopic ellipsometer operating in rotating compensator mode at an angle of incidence range of 60–80° (with a step of 2.5°) and spectral range of λ = 250–1000 nm. The data obtained was fitted with the CompleteEASE software using a multilayer model. Back side reflections (which limit the analytical performance when measuring on transparent substrates) were avoided by scratching the substrate backside. The complex refractive indices of all substrates were determined immediately after their activation using a superposition of Cauchy dispersion () and Urbach absorption tail (*k*(λ) = *k*_1_·*e*^*k*^_2_^(1240/λ–1240/λ^_b_^)^) functions, where *A*, *B*, *C*, *k*_1_, *k*_2_, and λ_b_ are adjustable parameters (Table S7). All reported thickness values are averages obtained from six independent measurements, expressed as mean ± standard deviation.

### X-ray Photoelectron Spectroscopy

X-ray photoelectron spectroscopy (XPS) measurements were carried out with a K-Alpha^+^ spectrometer (ThermoFisher Scientific, East Grinstead, UK). The samples were analyzed using a microfocused, monochromated Al Kα X-ray source at an angle of incidence of 30° (measured from the surface) and an emission angle normal to the surface. The kinetic energy of the electrons was measured using a 180° hemispherical energy analyzer operated in the constant analyzer energy mode at 200 and 50 eV pass energy for the survey and high-resolution spectra, respectively. To limit the X-ray-induced destruction of the thin polymer films and to maximize the signal-to-noise ratio, 20 individual points were measured within areas of 4 × 8 mm^2^. At each point, survey and high-resolution core-level spectra were measured. Spectral resolutions of 1.0 and 0.1 eV were used for the survey and high-resolution spectra, respectively. All reported XPS spectra are averages of the 20 individual measurements referenced to the C1s peak of hydrocarbons at 285.0 eV. Data acquisition and processing were performed using the Thermo Advantage software. The XPS spectra were fitted with Voigt profiles obtained by convolving Lorentzian and Gaussian functions to determine the amounts in atomic % of the individual chemical species present on the analyzed surfaces. For the analysis of the OTS and PFOTS layers, the ratios of the chemical species in the high-resolution spectra taken in the C 1s region were compared with the expected, i.e., theoretical values of chemical moieties that make up the OTS and PFOTS molecules. The unreacted OTS molecule has the overall chemical formula of CH_3_(CH_2_)_7_SiCl_3_. Based on the chemical formula of OTS, we can expect the following characteristic XPS contributions for the C 1s region: (a) C–Si (from the carbon atom bonded to the trichlorosilane group) and C–C (from the aliphatic CH_2_ and CH_3_ groups) contributions, with a characteristic (i.e., theoretical) quantitative ratio of 1:8 between the two contributions. Similarly, an unreacted PFOTS molecule has an overall chemical formula of CF_3_(CF_2_)_5_CH_2_CH_2_SiCl_3_. Considering the chemical formula of PFOTS, we can expect the following characteristic XPS signals for the C 1s region: (a) C–Si signal originating from the carbon atom bonded to the trichlorosilane group, a contribution of the CH_2_ group adjacent to the perfluorooctyl chain (i.e., C*–CF_2_), a contribution from the (CF_2_)_5_ chain, and a CF_3_ peak from the chain end. From the chemical formula PFOTS, we expect a characteristic quantitative ratio between the individual contributions constituting the C 1s spectrum of C–Si/C*–CF_2_/CF_2_/CF_3_ = 1:1:5:1. The close agreement between the theoretically expected and experimentally determined ratios can serve as a guideline for the chemical structure of the OTS and PFOTS layers. In the Cl 2p region, the presence of Cl–Si signals originating from the anchoring chlorosilane groups can be used to detect the presence of unreacted precursor molecules. The analyzer transmission function, Scofield sensitivity factors, and effective attenuation lengths (EALs) for photoelectrons were applied for quantification. EALs were calculated using the standard TPP-2 M formalism. The BE scale was controlled by the well-known position of the photoelectron C–C and C–H, C–O and C(=O)–O C 1s peaks of poly(ethylene terephthalate), and Cu 2p, Ag 3d, and Au 4f peaks of metallic Cu, Ag, and Au, respectively. The BE uncertainty of the reported measurements and analysis is in the range of ±0.1 eV.

### Dielectric Spectroscopy

A Novocontrol Alpha frequency analyzer consisting of a broadband dielectric converter and an active sample head was used. Samples were measured with two stainless steel electrodes with a diameter of 20 mm. A broad frequency range from 10^–2^ to 10^7^ Hz was employed. All measurements were conducted at an ambient temperature. The applied voltage had an amplitude of 1 V. More details about the measurement and the measurement uncertainty can be found in the Supporting Information. We measured each sample in five different areas. We used the average of five measurements to determine the permittivity of each glass. The measurement uncertainty was calculated by the standard deviation of the five measurements.

### ζ Potential of the Surface

ζ potential measurements, determined via the streaming potential, were carried out with a device from Anton Paar, SurPASS3 with a 10 × 10 clamping cell with a distance between the electrode and sample of 100 μm. We measured the ζ potential in KCl solutions at constant conductivity of 10 mS/m and different pH values to obtain the ζ potential titration curve; it is known that the ζ potential depends on both of them.^18,34^ HCl and KOH were used to adjust the pH. To determine the measurement uncertainty, we measured a sample at pH 5.5 at 10 different points. We used the standard deviation of these values to determine the measurement uncertainty. The uncertainty in the calculated ζ potential was determined by propagating the uncertainty.

### Sandblasting

Some sodium silicate glass slides were sandblasted in-house to enhance the surface roughness. The samples were placed in a box. Similar to work in a glovebox, the sandblasting gun was operated with gloves. The pressure was regulated with the sandblasting gun, while the distance between the sample and the sandblasting gun was approximately 20 cm. The gun was moved manually over the sample. The sand used (SiO_2_) had a grain size of 100–200 μm (AUER, Germany). The slides were treated with a pressure of 1.5 or 2 bar.

## Results and Discussion

**Figure 2:**
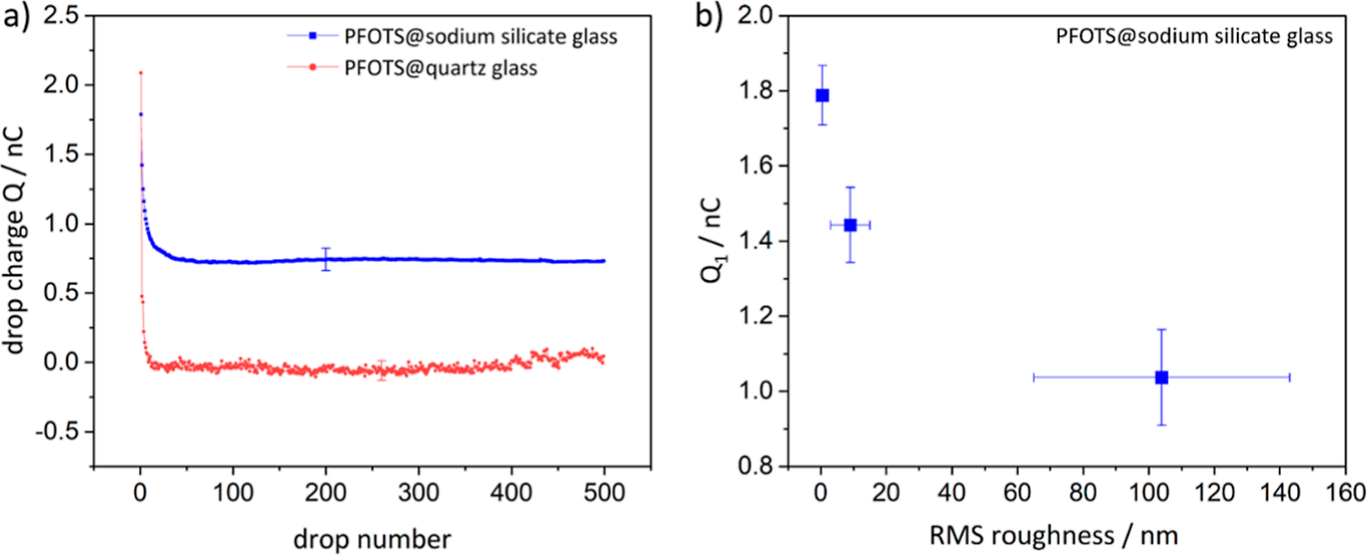
We measured the drop charge and assumed the counter charge to be on the surface. (a) Drop charge versus drop number for a series of 500 water drops of 45 μL moving down a surface at 50° tilt at a rate of one drop every 2 s. For the saturated drop charge, the average value of the drop charge of drop 200 until 500 was taken. The error bar indicates a typical variation from one sample to the next. (b) Drop charge of the first drop of the PFOTS@sodium silicate substrates is plotted versus the root-mean-square roughness.

For the evaluation, we used the drop charge of the first droplet, *Q*_1_, and the averaged drop charge *Q*_∞_ between droplets 200 and 500. We measured the drop charge and assumed that the counter charge to be on the surface. When measuring the drop charge for series of droplets sliding down at fixed interval time, the first droplet showed the maximum charge. Then the charge decreased to reach saturation (Figure 2a) in agreement with earlier reports.^8^ For PFOTS@sodium silicate glass, *Q*_∞_ was also positive. Quartz glass *Q*_∞_ became negatively charged typically after 20 droplets (Figure 2a).

Furthermore, *Q*_1_ decreased with increasing surface roughness (Figure 2b), which agrees with earlier observations.^16^

**Figure 3:**
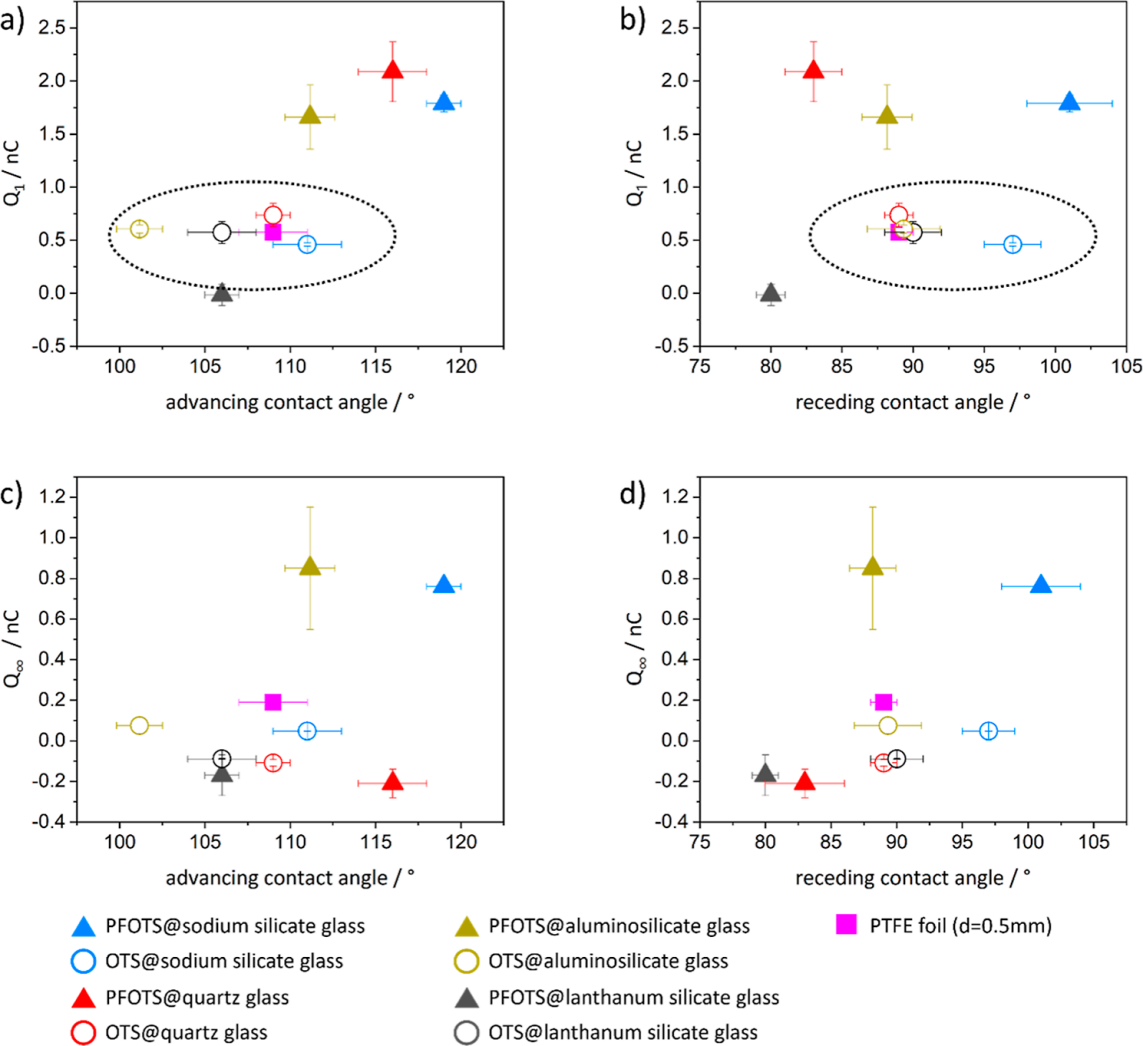
Drop charge is plotted against the static advancing and RCA of the OTS (hollow data points) and PFOTS (filled data points) surfaces. The drop charge of the first drop *Q*_1_ is plotted against the (a) ACA and (b) RCA. The saturated drop charge *Q*_∞_ is plotted against the (c) ACA and (d) RCA.

To check if the wetting properties affect the drop charge, we plotted *Q*_1_ and *Q*_∞_ against the static advancing contact angle (ACA) and the receding contact angle (RCA) (Figure 3). For both *Q*_1_ and *Q*_∞_, we did not observe a significant correlation between the drop charge and the wetting properties, at least not in the range considered.

It was observed that *Q*_1_ values on all OTS-coated substrates and on the PTFE foil were similar with *Q*_1_ ≈ 0.5–0.8 nC (dashed line, Figure 3a,b). In contrast, on the PFOTS-coated samples, *Q*_1_ depended on the substrate. *Q*_1_ values on PFOTS@quartz glass, PFOTS@sodium silicate glass, and PFOTS@aluminosilicate glass were in the range around 2.0 nC, while no drop charge was measured on PFOTS@lanthanum silicate glass.

When considering drop numbers 200–500 (Figure 3d), the drop charge on the PTFE foils decreased to roughly 30% of the first drop. For the OTS and PFOTS-coated samples, *Q*_∞_ depended on the substrate: For OTS@sodium silicate glass and OTS@aluminosilicate glass, *Q*_∞_ was 20–30% of *Q*_1_. For the OTS on quartz or lanthanum silicate glass, however, the droplet became even negatively charged (*Q*_∞_ ≈ −0.1 nC). For PFOTS@sodium silicate glass and PFOTS@aluminosilicate glass, *Q*_∞_ was 40–45% of *Q*_1_. For PFOTS on quartz or lanthanum silicate glass, the droplet also became negatively charged (*Q*_∞_ ≈ −0.2 nC).

For the further discussion of Figure 3, we analyzed the coating and substrate separately. Figure 3a (dashed circle) shows *Q*_1_ to have a similar magnitude in the case of a fully hydrogenated (OTS) or fully fluorinated (PTFE) surface. For the PFOTS (mixed hydrogenated/fluorinated)-coated substrates, *Q*_1_ was affected by the glass substrate. For glass, it is known that it is not as homogeneous as the network theory suggests.^27^ The atomic force microscopy (AFM) characterization of the coated and pristine sodium silicate glass showed an inhomogeneous surface (Figures S2 and S3). Since the original sodium glass surface was inhomogeneous, we exclude that we leached the surface during the cleaning step or during the drop charge experiments. Only for the lanthanum glass, we observed leaching after the treatment with HCl and MeOH (Figure S1). Therefore, we did not use this cleaning step for the lanthanum glass. Therefore, we exclude that leaching influenced the drop charge experiment. Furthermore, we exclude that ion diffusion from the substrate to droplet affects the drop charge, because ion migration in alkali glass was observed experimentally only at temperatures above 100 °C.^35^ However, this raises the question to the extent to which the homogeneity of the coating is affected by the glass substrate.^31^ The chemical identification of the observed inhomogeneity remains a challenge. KPFM analysis was not done due to the low conductivity and the thickness (1 mm) of the glass slides. For Raman spectroscopy, the lateral resolution is around 1 μm. We scanned the coated sodium silicate surface with AFM at a scan size of 1 μm × 1 μm (Figure S7). Even at this scan size, we observed the inhomogeneity and we could not use Raman spectroscopy to investigate the homogeneity of the coating, because it is below the resolution limit of Raman spectroscopy. Due to the inhomogeneity properties of the glass, we prepared all samples at least three times and measured the drop charge on all samples three times. We used ellipsometry to characterize the thickness and to check how far the coatings differ from each other. The theoretical layer length of a OTS or PFOTS molecule oriented perpendicular to three oxygen atoms of the substrate was around 1 nm. These values were calculated with the open source software Avogadro (force field: universal force field, Figure S8).^36^ For the OTS coatings, we indeed measured a layer thickness in the range of a calculated monolayer on all glass substrates (∼1 nm, Table 1). In contrast, the thickness of the PFOTS layer differed from substrate to substrate. The thickness increased from the calculated values of a monolayer the PFOTS@quartz glass (1.1 ± 0.1 nm) and PFOTS@aluminosilicate glass (1.1 ± 0.4 nm) to values, indicating the formation of a multilayer siloxane network of 4.9 ± 1.8 and 5.6 ± 0.5 nm on PFOTS@sodium silicate and PFOTS@lanthanum silicate glass substrates, respectively.

**Figure 4:**
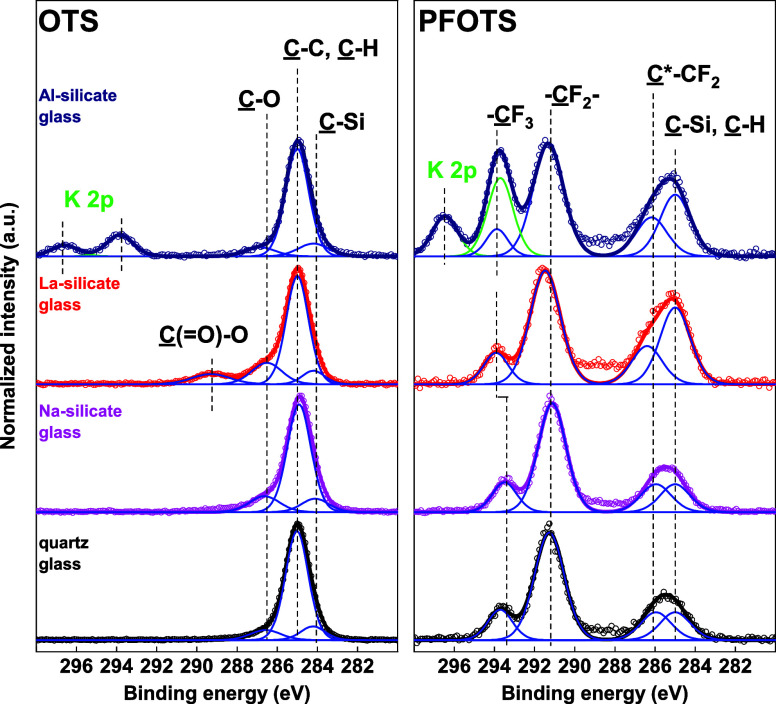
High-resolution XPS spectra in the C 1s region of PFOTS and OTS layers formed on quartz, sodium silicate, and lanthanum silicate glass. Measured spectra (open cycles) were deconvoluted with individual contributions (blue lines). The resulting fitted envelopes of the PFOTS and OTS layers are presented with thick black, magenta, and red lines for the respective quartz, sodium silicate, and lanthanum silicate glass substrates.

The ellipsometry results were corroborated by XPS (Figure 4). High-resolution C 1s XPS spectra of all OTS layers could be deconvoluted with a C–Si contribution at 284.2 ± 0.1 eV and a dominating C–C moiety at 285.0 eV arising from the aliphatic CH_2_ and CH_3_ groups. The chemical formula of OTS (CH_3_(CH_2_)_7_SiCl_3_) suggests a peak ratio of 1:8 between C–Si/C–C moieties. The C–Si/C–C amount ratios between the moieties on OTS layers formed on quartz, sodium silicate, lanthanum silicate, and aluminosilicate glass substrates were determined from the XPS surface data as 1.0:7.9, 1.0:6.9, 1.0:8.0, and 1.0:6:6, respectively, and thus agree with the expected values (Table S8). The observed minor contributions at 286.5 ± 0.2 and 289.3 ± 0.2 eV originate from various adversely physiosorbed C–O hydroxyl or ether and C(=O)–O ester or organic acid species, respectively. In summary, ellipsometry and XPS show that OTS substrates have a coating with comparable chemical anchoring and a layer thickness of ≈1 nm, independent of the type of glass (Tables 1 and S8). Therefore, we assume that for the OTS coating, the surface coverages on all substrates are in the same range.

High-resolution C 1s XPS spectra of all PFOTS layers have been deconvoluted with a C–Si contribution at 285.0 eV (strongly overlapped with C–C contributions of adventitious carbon contamination), a secondary shift C*–CF_2_ peak at 286.0 ± 0.1 eV, a dominating CF_2_ peak at 291.1 ± 0.1 eV, and a CF_3_ peak at 293.5 ± 0.1 eV. In comparison, for PFOTS surfaces the C–Si appeared at higher binding energy (285.0 eV, most probably due to the higher electronegativity of the (1H,1H,2H,2H-perfluorooctyl) chain in comparison to the n-octyl aliphatic chain. Based on the chemical formula of the PFOTS layer (CF_3_(CF_2_)_5_CH_2_CH_2_SiCl_3_), a chemically bound molecule on the substrates would lead to the expected peak ratios between the individual contributions of C–Si: C*–CF_2_/CF_2_/CF_3_ = 1:1:5:1. The quantitative ratios between the C–Si: C*–CF_2_/CF_2_/CF_3_ moieties on PFOTS layers formed on quartz, sodium silicate, lanthanum silicate, and aluminosilicate glasses were determined as 1.2:1.1:4.4:1.0, 1.3:1.0:4.4:1.0, 3.0:1.5:4.5:1.0, and 3.5:2.3:6.3:1.0, respectively (Table S8). Neglecting the adventitious carbon contamination contributions, the analysis of the C 1s spectra points to the attained PFOTS chemical structure. The decrease (in atom %) of the chemical species arising from the substrates and the concomitant increase of the carbon and organic fluorine content in the surface composition of PFOTS modified glasses indicate a different thickness of the siloxane layers (Table S8). Here, higher amounts of PFOTS were observed on sodium silicate and lanthanum silicate glass than on quartz glass and aluminosilicate glass. The different layer thicknesses are in agreement with the ellipsometry measurements (Table 1). Notably, all PFOTS and OTS layers lack Cl 2p contributions arising from Si–Cl (Table S8 and Figure S9), which further proves the complete conversion of the PFOTS and OTS molecules to single or multilayer siloxane structures.

In summary, the consistent results of ellipsometry and XPS show that all of the substrates coated with OTS and PFOTS have a coating with comparable chemical anchoring but different thickness.

**Figure 5:**
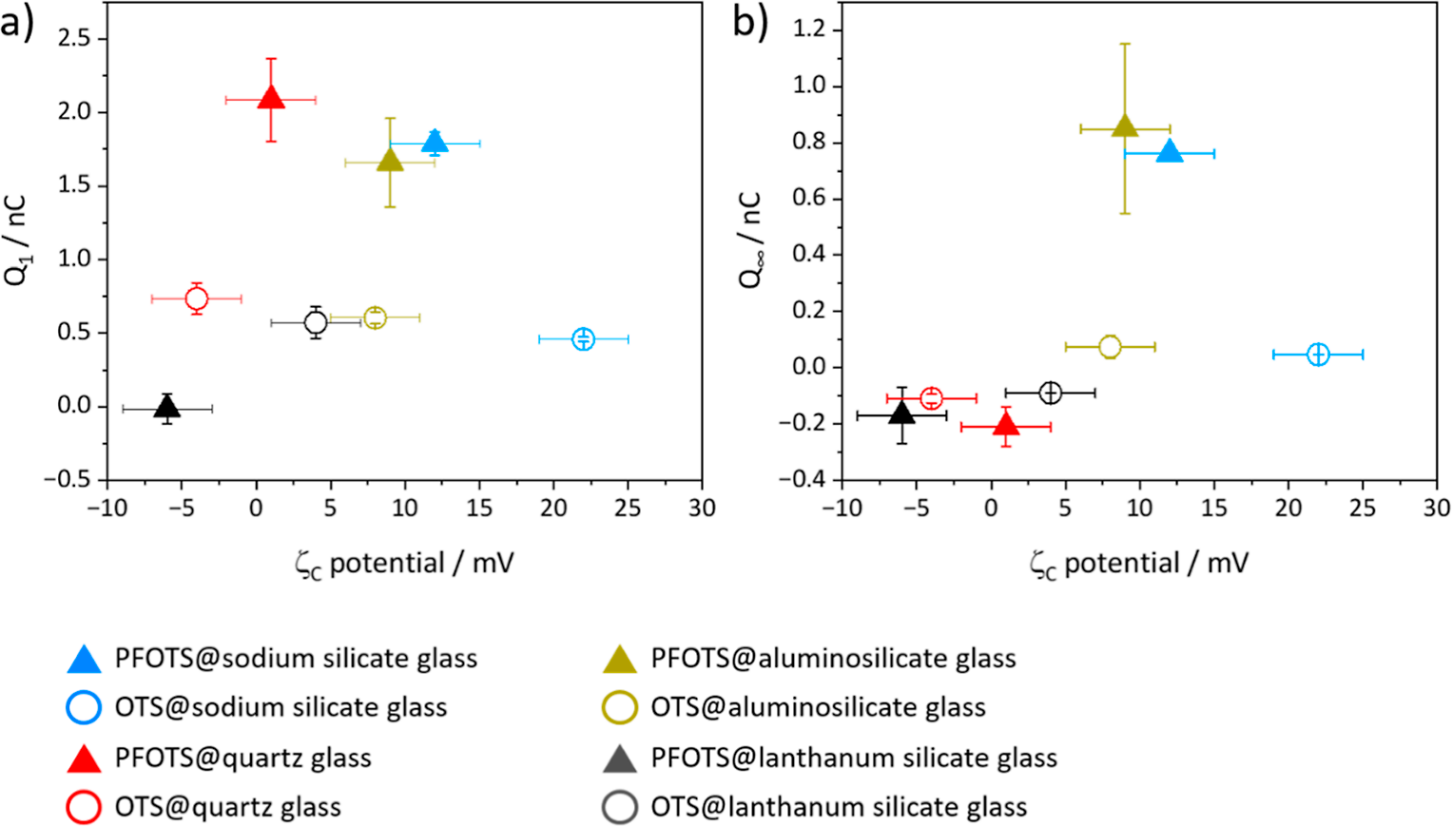
We calculated the ζ_c_ potential of the coating by the assumption that ζ potentials are addable. (ζ_c&s_ potential = ζ_s_ potential + ζ_c_ potential). (a) *Q*_1_ is plotted against ζ_c_ potential and (b) *Q*_∞_ is plotted against ζ_c_ potential.

To test how far the different surface coverage affects charge separation, we measured the ζ potentials of the coated (ζ_c&s_Table 1) and noncoated substrates (ζ_s_Table 2. In all cases, the ζ potentials decreased with pH from −19 to +17 mV (pH 3.5) to ≈ −60 mV (pH 10) (Figure S10). Assuming that the individual ζ potentials are summable, we calculated the ζ potential of the OTS and PFOTS coating (ζ_c_) by taking the difference between the ζ_c&s_ potential of the substrate with each coating and the corresponding ζ_s_ potential of the substrates ζ_c_ ζ_c&s_ – ζ_s_)·For the calculation, we used the ζ potential of a pH value of around 5.5 (corresponding to the pH value of the DI water used at the drop charge measurements). Contrary to expectations (same coating), the ζ_c_ potentials of the OTS and PFOTS coating differ from substrate to substrate (Figure 5). This agrees with the previous ellipsometry and XPS results and shows that the coatings differ from substrate to substrate. Furthermore, there was no correlation between ζ_c_ potential and *Q*_1_ or *Q*_∞_ (Figure 5). We can state the following regarding the coating: For *Q*_1_, the degree of hydrogenation or fluorination is one parameter that influences slide electrification. For a fully hydrogenated or fully fluorinated surface such as an OTS coated surface or a PTFE surface, *Q*_1_ was in the same order of magnitude. With respect to the acid base model of slide electrification,^11^ this agrees with a reported DFT calculation that the absorption energy of a water molecule on a hydrophobic surface is related to the degree of fluorination/hydrogenation.^22^

**Table 2:** Density (ρ), ζ_substrate_ Potential, Mean Molecular Weight (*M*_mean_), Permittivity (ε_r_), and Polarizability (α) of the Glass Substrates. *N*_A_ is the Avogadro Constant. ε_0_ is the Dielectric Permittivity of Vacuum. The ζ Potential Value Corresponds to the Measured ζ Potential at a pH of 5.5

substrate	ρ/g cm^–3^	ζ_substrate_/mV	*M*_mean_/g mol^–1^	ε_r_/A s^–1^ V m^–1^	α × *N*_A_ × ε_0_^–1^/cm^3^
quartz glass	2 ± 0.5	–16 ± 2	60	3.65 ± 0.01	354 ± 1
sodium silicate glass	2 ± 0.5	–26 ± 2	60	6.53 ± 0.06	641 ± 1
lanthanum silicate glass	3 ± 0.3	–17 ± 2	133	10.95 ± 0.06	1106 ± 1
aluminosilicate glass	2.5^a^	–26 ± 2	70.2	7.7^a^	819 ± 1

a Data provided by Schott AG, Mainz.

Furthermore, we confirmed that the thickness of the coating was substrate-dependent.^31^ Since neither the calculated ζ_c_ potentials of the coating nor the layer thickness correlate with *Q*_1_ or *Q*_∞_, we assume that slide electrification is influenced by the substrate in addition to the coating. On the other hand, the results showed us that there could be an interaction between the surface coating and the substrate.

To analyze the influence of the substrate on slide electrification, we calculated the polarizability α (Table 2) with the Clausius–Mossotti equation.^28^
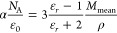
1

*M*_mean_ is the mean molecular weight of the glass substrate (Tables S1–S4). The density ρ was calculated using the measured weight and volume of each substrate (Table S9). N_A_ is the Avogadro constant, ε_0_ is the vacuum dielectric permittivity, and ε_r_ is the relative dielectric permittivity of the substrate (Figure S9). The errors displayed in Table 2 of the density and polarizability were calculated with Gaussian error propagation. The measured weight and the length, width, and thickness of each glass substrate are listed in Table S9. In contrast to the other glasses, we had to cut the aluminosilicate glass ourselves. Due to its small thickness (*d* = 0.330 mm), we therefore used the density and permittivity provided by the supplier. For this reason, we do not report an uncertainty of this density or the permittivity.

**Figure 6:**
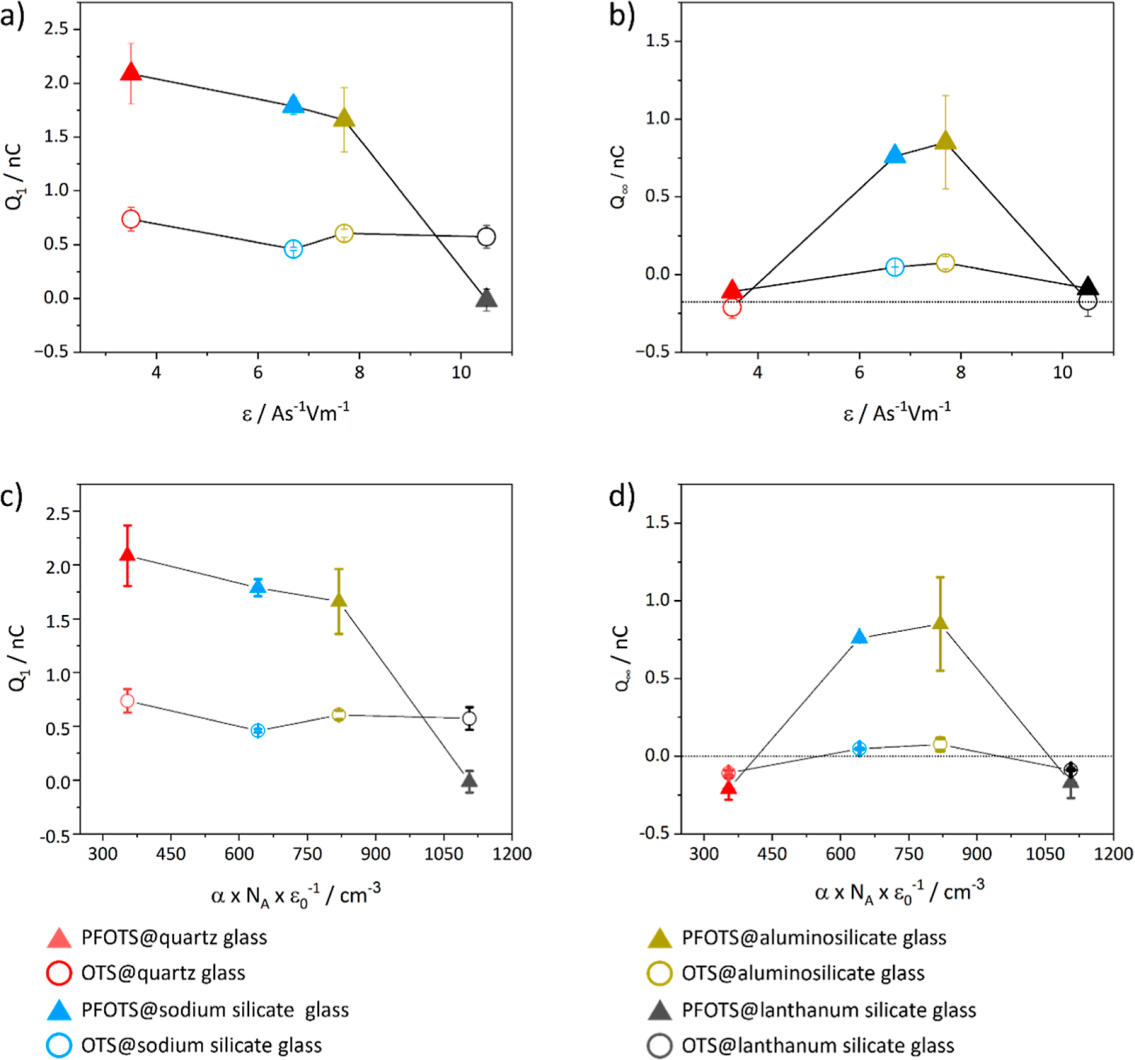
Drop charge plotted against polarizability α and permittivity ε. (a) Drop charge of the first drop *Q*_1_ plotted against ε.(b) Saturated drop charge *Q*_∞_ against ε.(c) Drop charge of the first drop *Q*_1_ plotted against α.(d) Saturated drop charge *Q*_∞_ against α.

When plotting *Q*_1_ and *Q*_∞_ against α or ε_r_ for the OTS coated substrates, we found no correlation (Figure 6). *Q*_1_ was in all samples around 0.5 nC. For the PFOTS coated substrates, we observed that *Q*_1_ decreases with increasing α or ε_r_ (Figure 6a,c). *Q*_∞_ did not correlate with α or ε_r_ (Figure 6b,d). However, we observed that the *Q*_∞_ values of the OTS- and PFOTS-coated sodium silicate glass and aluminosilicate glass had a positive drop charge. *Q*_∞_ was negative in all other samples independent of the coating or substrate. The ζ_s_ potentials of the bare sodium silicate and aluminosilicate glasses are in a similar range, especially in the pH range of 3 to 6 (Figure S10). Assuming that the ζ potential is related to the hard soft acid–base (HSAB) principle, we analyzed the chemical composition of the substrates.^37^ A comparison of the chemical composition of the surface (Table S8) and the bulk (Tables S2 and S4) of the two glasses revealed that both glasses contain magnesium, sodium, aluminum, and potassium. Furthermore, we saw that the distribution of the components on the surface and bulk are inhomogeneous.^27^

**Figure 7:**
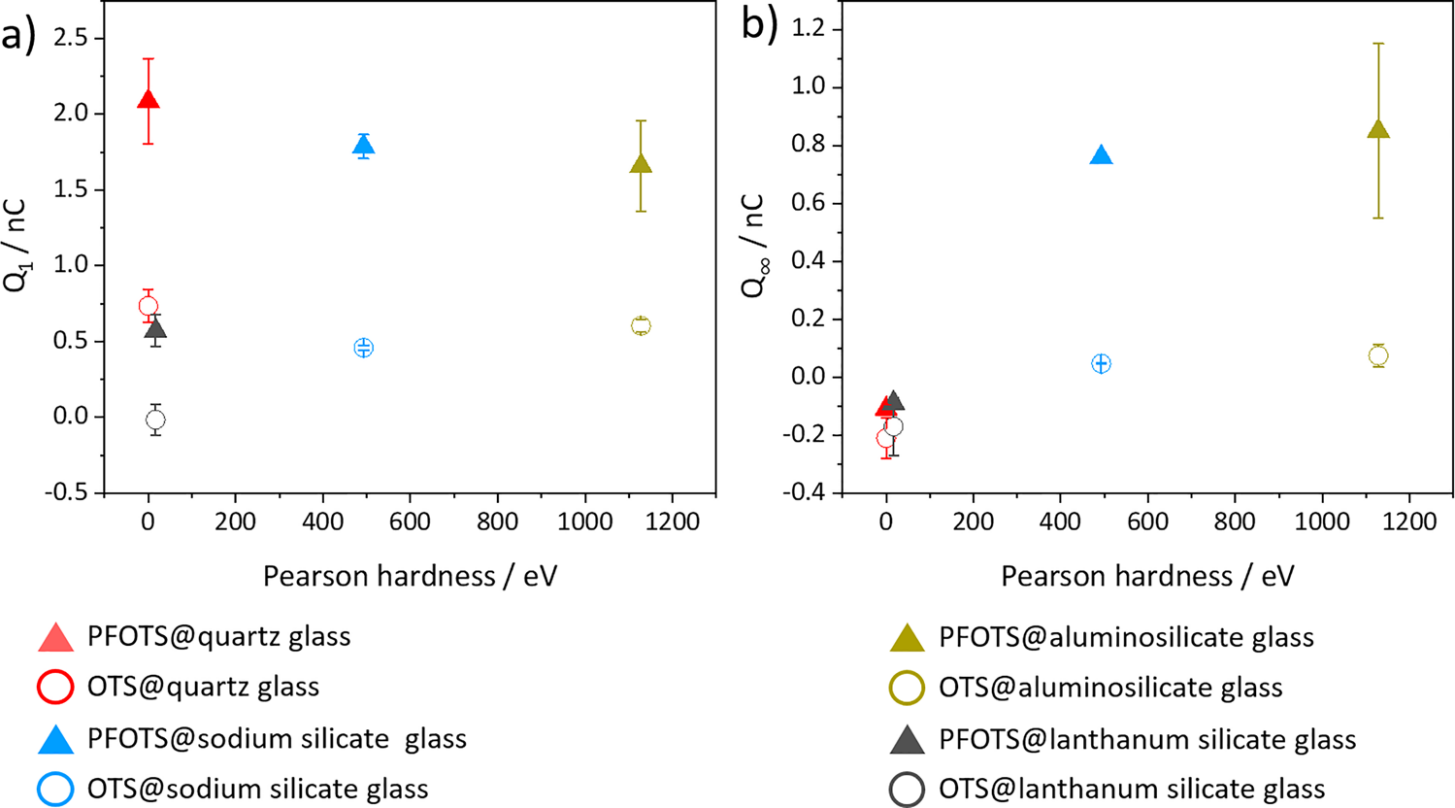
Drop charge plotted against the Pearson hardness. (a) Drop charge of the first drop *Q*_1_ plotted against the Pearson hardness.(b) Saturated drop charge *Q*_∞_ against the Pearson hardness.

As described in the previous paragraph, *Q*_∞_ (Figure 2a,b) is smaller than *Q*_1_. We assumed that the adsorbed hydroxide ions on the surface can interact with the oxonium ions or protons of the following droplet (Brønsted acid–base reaction^26^). It is known that the acidity or basicity of a substrate can be changed by the incorporation of special elements such as aluminum.^38^ The acidity or basicity of a chemical species can be calculated by using the HSAB concept (involving the orbital energies). The Pearson concept is a system for classifying acids and bases in chemistry based on their specific properties. Hard acids and bases are characterized by small size, high charge, and low polarizability, while soft acids and bases are larger and have a lower charge and higher polarizability. This concept helps to predict the stability of compounds, the outcome of chemical reactions, and the formation of compounds by associating hard acids with hard bases and soft acids with soft bases.^39^ If we compare the calculated acidity (Pearson hardness) of sodium (21 eV), magnesium (33 eV), and aluminum (46 eV), we see that magnesium and aluminum have a high acidity.^37,40^ The quantities of these elements in the different substrates are summarized in Table 3. Thus, the positive *Q*_∞_ value of the aluminosilicate and sodium silicate glass could be attributed to the acidity of aluminum, sodium, and/or magnesium. The other components had a Pearson hardness below 20 eV; we just summarized species with a Pearson hardness above 20 eV, because we assumed that this is the hardness factor, which is important for the interaction with the hard hydroxide ion (Pearson hardness of 5.7 eV).^37,40,41^ We plotted *Q*_1_ and *Q*_∞_ against the Pearson hardness of the substrates (Figure 7). There is no obvious correlation for *Q*_1_ between the drop charge and Pearson hardness, but *Q*_∞_ increases with increasing Pearson hardness. Therefore, we assumed that the Pearson hardness in the saturated state affects the acid–base equilibrium between the droplet and the surface.

**Table 3:** Species, Fraction of the Species, and the Pearson Hardness of Each Species of the Different

substrate	species	fraction/%	Pearson hardness η/eV	sum Pearson hardness η/eV
sodium silicate glass	Al^3+^	1.25	46	493
	Na^+^	13.99	21	
	Mg^2+^	4.32	33	
aluminosilicate glass	Al^3+^	16.24	46	1128
	Na^+^	12.32	21	
	Mg^2+^	3.73	33	
lanthanum silicate glass	Al^3+^	0.38	46	17
quartz glass				0

## Conclusions

We were able to expand our understanding of slide electrification by analyzing the influence of the surface and substrate chemistry. We show that slide electrification is influenced by the surface and substrate chemistry. In a series of droplets sliding over a hydrophobic surface, the drop charge of the first drop *Q*_1_ of a given series and the drop charge in the saturated state *Q*_∞_ (drop charge after around 100 drops) cannot be described by one parameter. The charge of the first drop, *Q*_1_ is influenced by the degree of hydrogenation or fluorination of the surface. For a fully hydrogenated or fluorinated glass surface, the influence of the substrate is negligible. For a mixed hydrogenated/fluorinated glass surface, *Q*_1_ is influenced by the substrate. Here *Q*_1_ decreased with increasing permittivity or polarizability of the substrate. In the saturated state, both the permittivity and the polarizability did not correlate with the saturated drop charge *Q*_∞_, regardless of the degree of hydrogenation or fluorination of the glass surface. *Q*_∞_ correlates with the Pearson hardness of the substrate. With increasing Pearson hardness, *Q*_∞_ is positive. The calculated ζ potential of the coating correlates analogously to the Pearson hardness only with the *Q*_∞_. In this way, we showed that the substrate chemistry plays an important role for the formation of the drop charge in the saturated state, *Q*_∞_. By varying the substrate (e.g., by incorporating Al^3+^), we demonstrate the impact of the substrate chemistry by exploiting the HSAB property (Pearson hardness) of Al^3+^. We extended the model of the acid–base reaction between the droplet and surface by the HSAB model. As the Pearson hardness is a property of the substrate related to its acidity, the origin of the charge is the interaction of OH^–^ and H^+^ with the substrate rather than the coating via acid–base reactions, i.e., the role of the substrate seems to be dominant. Therefore, future efforts to increase the drop charge in future applications should focus on manipulation of the substrate chemistry.

## Supporting Information Available

Chemical composition of the different glass substrates, etching of the lanthanum silicate glass, analysis of the silanization, AFM analysis of all surfaces, description of the droplet charge measurement and all droplet charge measurements on all substrates, all measured parameters of the spectroscopic ellipsometry, XPS surface analysis, measured ζ-potential curve of all coated and noncoated substrates, parameters used to calculate the density of the glass substrates, and dielectric spectra of the glass substrates (PDF)
