# Supplementary material for: How Surface and Substrate Chemistry Affect Slide Electrification
Source: J Am Chem Soc. 2024 Apr 2;146(14):10073–83. doi: 10.1021/jacs.4c01015 (PMC11009953; doi:10.1021/jacs.4c01015)
Supplement: Supplementary file 1 — ja4c01015_si_001.pdf [file ja4c01015_si_001.pdf]

Supporting Information for

How Surface and Substrate Chemistry Affect Slide Electrification

Benjamin Leibauer[1], Ognen Pop-Georgievski[2], Mariana D. Sosa[1], Yun Dong[1], Wolfgang Tremel[3], Hans-Jürgen Butt[1], Werner Steffen[1*]

[1] Max Planck Institute for Polymer Research, Ackermannweg 10, 55128 Mainz, Germany

[2] Institute of Macromolecular Chemistry, Heyrovského nám. 2, 162 00 Prague, Czech Republic

[3] Johannes-Gutenberg University, Chemistry Department, Duesbergweg 10-14, 55128 Mainz, Germany

* Corresponding author: Prof. Dr. Werner Steffen, steffen@mpip-mainz.mpg.de

Table of Contents

Item	Title	Page
Table S1	Chemical composition of the quartz glass slides	S3
Table S2	Chemical composition of sodium silicate glass	S3
Table S3	Chemical composition of lanthanum silicate glass	S3
Table S4	Chemical composition of aluminosilicate glass	S4
Figure S1	AFM topography of lanthanum silicate glass before and after HCl/methanol treatment	S5
Table S5	XPS analysis of the chemcical composition of lanthanum glass before treatment with HCl/methanol	S6
Table S6	XPS analysis of the chemcical composition of lanthanum glass after treatment with HCl/methanol	S6
Figure S2	AFM characterization of the PFOTS-coating via CVD	S7
Figure S3	AFM topography of all coated surfaces and PTFE foil	S8
Figure S4	Calculation of the drop charge	S9
Figure S5	Measured all drop charge on PFOTS@aluminosilicate glass as example to show the evaluation of the drop charge experiments	S10
Figure S6	Averaged drop charge of all samples	S10
Table S7	Parameter of Cauchy dispersion and Urbach absorption tail from the fitted ellipsometry data	S11
Figure S7	AFM images of PFOTS@sodium silicate glass at a scan size of 1 μm x 1 μm	S11
Figure S8	Model structure and length of a PFOTS/OTS molecule on glass	S12
Table S8	Atom % of chemical moieties present on the surfaces of quartz, sodium silicate and lanthanum silicate glasses before and after modification with PFTOS and OTS	S13-S14
Figure S9	High-resolution XPS spectra in the Cl 2p region of PFOTS and OTS coated glass substrates	S15
Figure S10	ζ potentials of coated and non-coated substrates	S16
Table S9	Weight, length, width and calculated volume of different glass substrates	S17
Figure S11	Dielectric spectra with corresponding permittivities of different glass substrates	S18

Glass Substrates

Table S1. Chemical composition of the quartz glass slides taken from the manufacturer information Thermo Fisher Scientific, USA.

Chemical component	Molar mass / g·mol^{-1}	Fraction / %	M_{mean} / g·mol^{-1}
SiO_2	60.08	99.99	60.08

The chemical composition of sodium silicate glass was analyzed by the IGR Institut für Glas- und Rohstofftechnologie, Göttingen, Germany.

Table S2. Chemical components of sodium silicate glass / Menzel microscope glass slides (Thermo Fisher scientific Gerhard Menzel B.V. & Co. KG, Germany).

Chemical component	Molar mass / g·mol^{-1}	Fraction / %	M_{mean} / g·mol^{-1}
SiO_2	60.08	72.90	60.09
MgO	40.30	4.32	
Na_2O	61.98	13.99	
Al_2O_3	101.96	1.25	
K_2O	94.20	0.93	
CaO	56.08	6.26	
SO_3	80.06	0.28	

The chemical composition of lanthanum silicate glass was analyzed by IGR Institut für Glas- und Rohstofftechnologie, Göttingen, Germany.

Table S3. Chemical components of lanthanum silicate glass / N-LASF45 microscope slides (Schott AG, Mainz, Germany).

Chemical component	Molar mass / g·mol^{-1}	Fraction / %	M_{mean} / g·mol^{-1}
SiO_2	60.08	24.9	107.02
ZnO	81.39	5.8	
La_2O_3	325.81	14.2	
Al_2O_3	101.96	0.38	
Nb_2O_3	108.9	16.1	
TiO_2	79.86	9.3	
CaO	56.08	24.6	
SrO	103.62	0.16	
ZrO_2	123.22	4.5	

The chemical composition of the aluminosilicate glass was analyzed by IGR Institut für Glas- und Rohstofftechnologie, Göttingen, Germany.

Table S4. Chemical components of the aluminosilicatesilicate glass / AS 87® eco thin glass (Schott AG, Mainz, Germany).

Chemical component	Molar mass / g·mol^{-1}	fraction / %	M_{mean} / g·mol^{-1}
SiO_2	60.08	60.00	70.21
MgO	40.30	3.73	
Na_2O	61.98	12.32	
Al_2O_3	101.96	16.24	
K_2O	94.20	4.02	
CaO	56.08	0.13	
SO_3	80.06	0.28	
ZrO_2	123.22	0.87	
TiO_2	79.86	0.02	
SnO_2	150.71	0.07	
Fe_2O_3	159.69	0.02	

Etching of Lanthanum Silicate Glass

After treatment with methanol and hydrochloric acid (37%) (ratio 1:1), the lanthanum silicate glass showed a blue reflection indicating a change of the surface composition. The AFM analysis revealed that the lanthanum silicate glass was etched (Figure S1). The XPS surface analysis showed that not only the roughness, but also the chemical surface composition of the lanthanum silicate glass had changed (Table S5 and table S6).

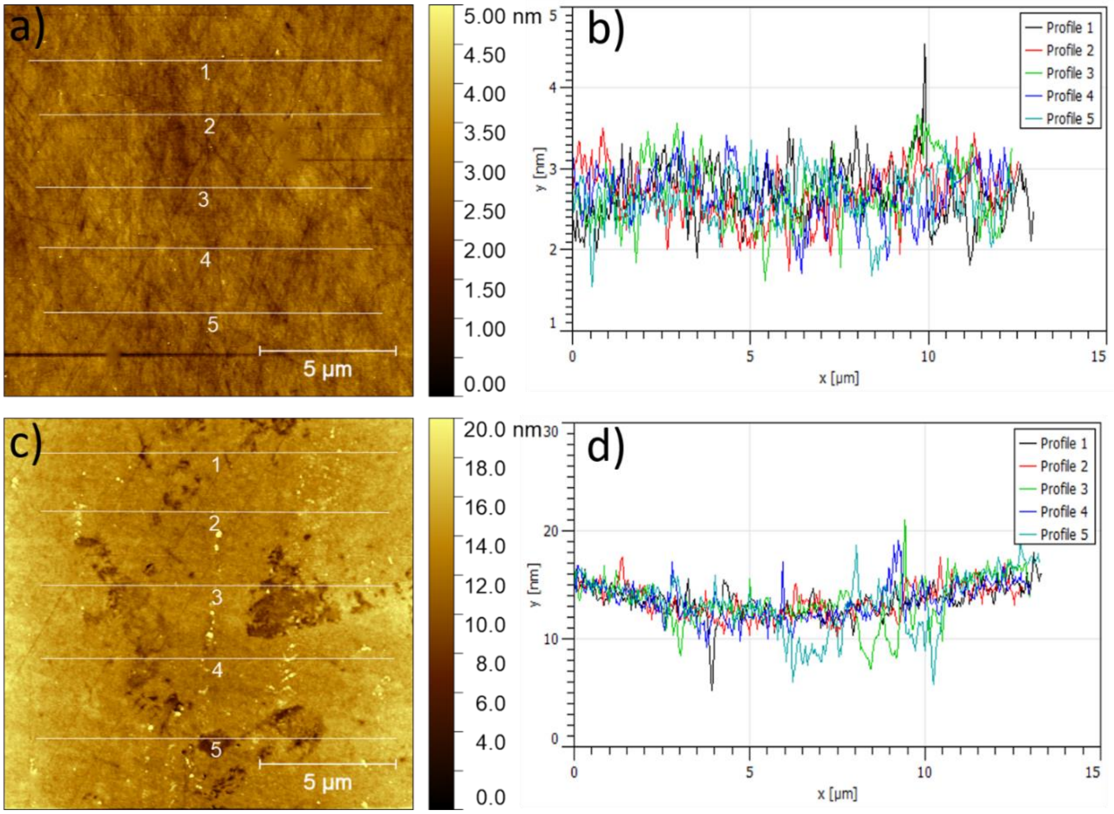

Figure S1. The AFM topography of lanthanum silicate glass was measured at a scan size of 15 × 15 µm (tapping mode). a) AFM topography of lanthanum silicate glass before treatment with HCl/methanol (ratio 1:1, 30 min). b) Height profiles of the non HCl/methanol treated surface. c) AFM topography of lanthanum silicate glass after treatment with HCl/methanol. d) Height profiles of the HCl/methanol treated surface. Height profiles were drawn and measured through each AFM image at five different positions.

Table S5. Chemical surface composition of lanthanum silicate glass before treatment with methanol and hydrochloric acid (37%) (ratio 1:1) (see Substrates and Surface treatment). The analysis was done using XPS.

Element specific peak	Amount / atomic %
Al 2p	0.3
Si 2p	13.1
B 1s	0.5
Nb 3d5	2.6
C 1s C-C	3.1
Zr 3p3	0.6
Ca 2p3	7.1
Ti 2p3	2.9
O 1s	63.2
F 1s	2.7
La 3d5	1.9
Zn 2p	2.0

Table S6. Chemical surface composition of lanthanum silicate glass before treatment with methanol and hydrochloric acid (37%) (ratio 1:1). The analysis was done using XPS (see Substrates and Surface Treatment).

Element specific peak	Amount / atomic %
Al 2p	0.3
Si 2p	32.6
Nb 3d5	0.2
C 1s C-C	1.7
Zr 3p3	0.2
Sb 3d5	0.3
Ti 2p3	0.1
O 1s	64.7

Silanization of Glass Substrates *via* CVD

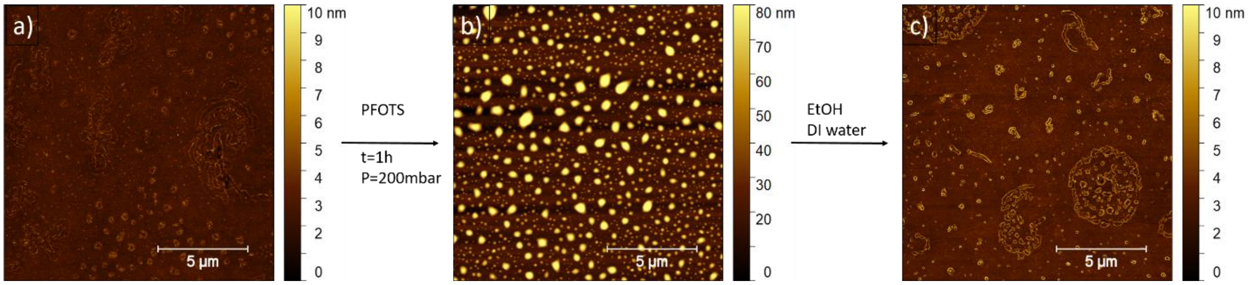

Figure S2. AFM analysis of sodium silicate glass after fluorination. AFM measurements were done in tapping mode at a scan size of 15x15 µm. a) Pristine sodium silicate glass. b) PFOTS@sodium silicate glass with side products. c) PFOTS@sodium silicate glass after rinsing with DI water and ethanol. The thickness of the PFOTS layer can not be detected by AFM. There is no distinction between clean and coated glass.

AFM Characterization of Coated Glass Substrates

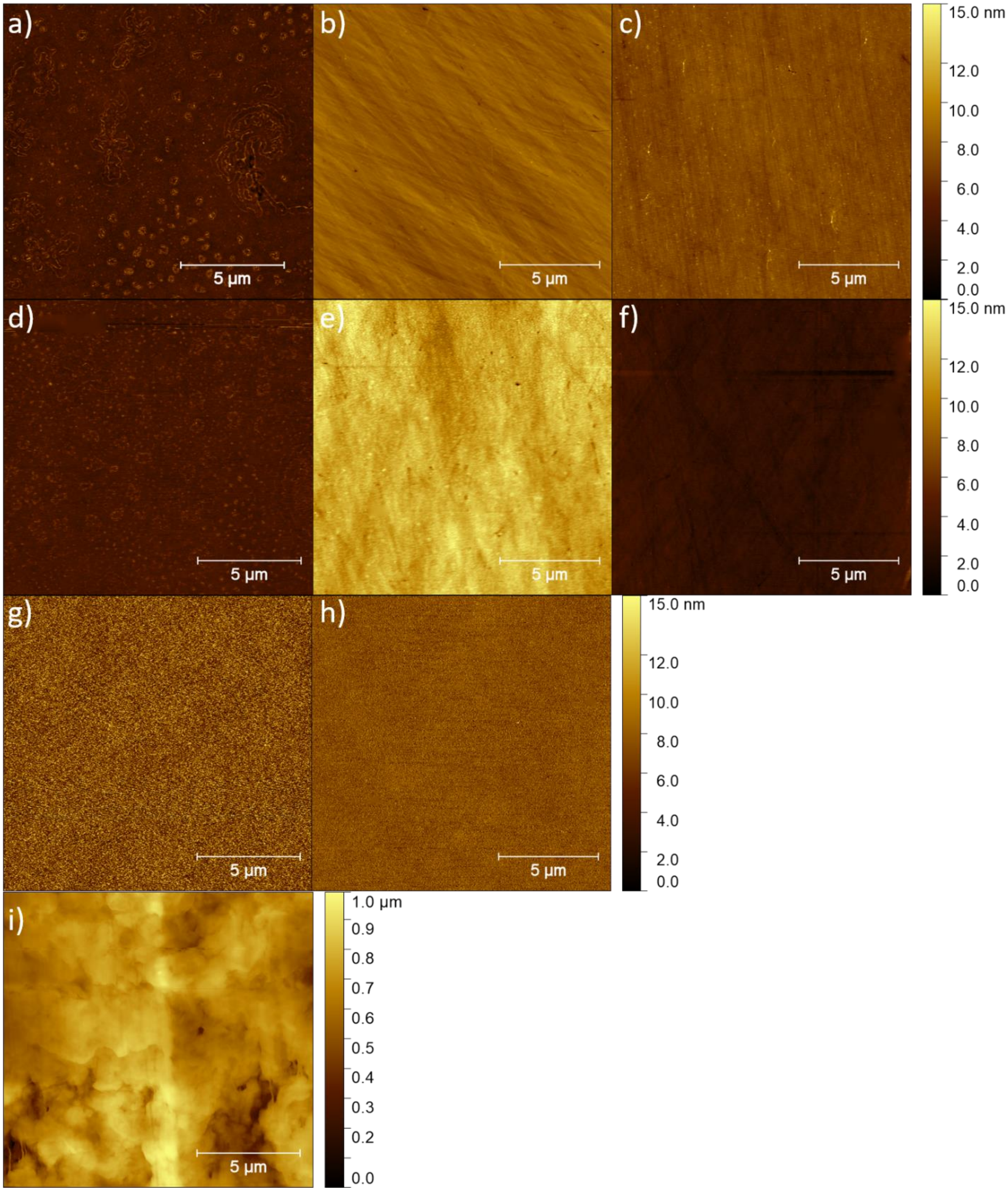

Figure S3. AFM measurements were done in tapping mode at a scan size of 15x15 µm. a) PFOTS@sodium silicate glass, b) PFOTS@quartz glass, c) PFOTS@lanthanum silicate glass, d) OTS@sodium silicate glass, e) OTS@quartz glass, f) OTS@lanthanum silicate glass, g) OTS@alumino silicate glass, h) PFOTS@aluminosilicate glass, i) PTFE foil.

Drop Charge

With our experimental setup the charge of the sliding droplets was obtained as follows. With the help of a current amplifier we measured the current of a sliding droplet. We obtained the discharge time (t^0 and t^1) of the droplet at the probe. By integrating the current over the discharge time ($\int_{t^0}^{t^1} I\,dt$) we calculated the charge of each droplet. For the analysis, we plotted the drop charge against the drop number.

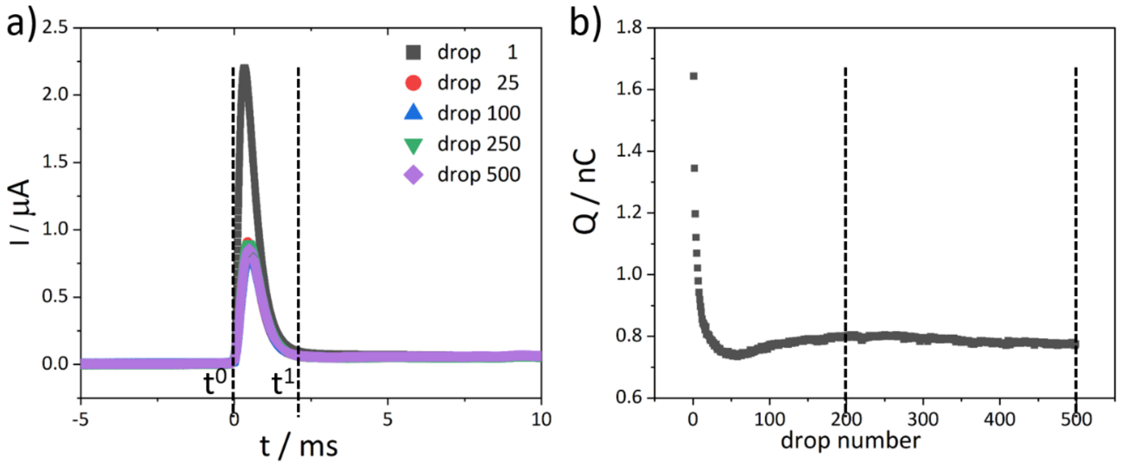

Figure S4. a) Current signal of a sliding droplet and discharge time of the droplet at the probe. By integration of the current over the discharge time (t^0, t^1) we calculated the drop charge of each droplet. b) Calculated drop charge against the drop number. With increasing drop number the drop charge decreases. We used the average of the drop charge between droplet 200 and 500 as saturated drop charge $Q\infty$.

Drop Charge evaluation

The drop charge experiments were evaluated as follows. We prepared each sample three times. We measured the drop charge at three different areas of each sample (Figure S5 a). Then we calculated the average value for each drop charge for with respect to the corresponding drop number (Figure S5 b). We used the standard deviation to determine the measurement uncertainty of each drop charge. For the measurment uncertainty in the saturated state, we used the standard deviation of the drop charge of drop number 250 (Figure S5 b). For the better overview we shifted this value from drop number 200 to 500 by an interval of 30 in Figure S6. Also for the better overview we did not plot the error bar of the first drop in Figure S6.

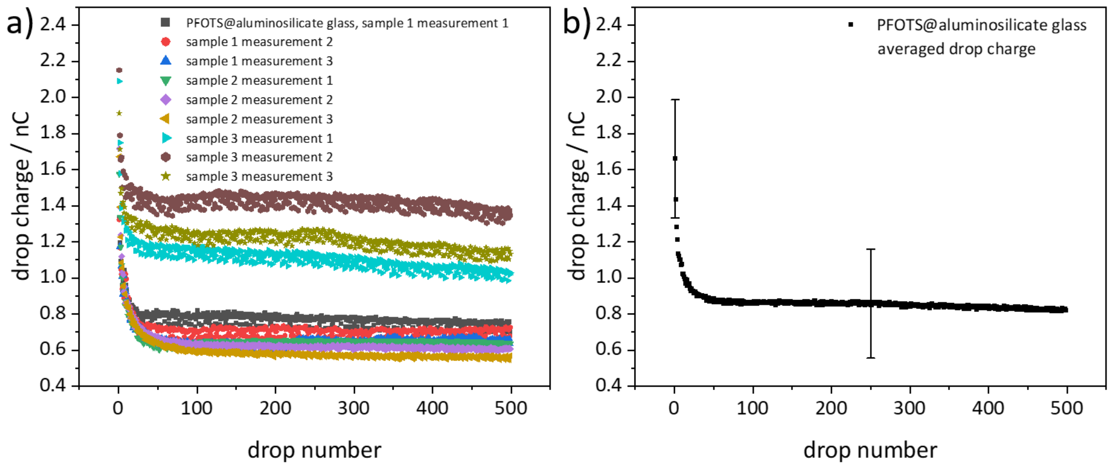

Figure S5. a) The measured drop charge of all three PFOTS@aluminosilicate glass samples. The drop charge is plotted against the corresponding drop number. b) We have calculated the mean value from the measured drop charges of the corresponding drop numbers and plotted the mean value against the respective drop numbers. The uncertainty of the drop charge was determined using standard deviation. For the saturated drop charge we used the uncertainty of the drop charge of drop number 250.

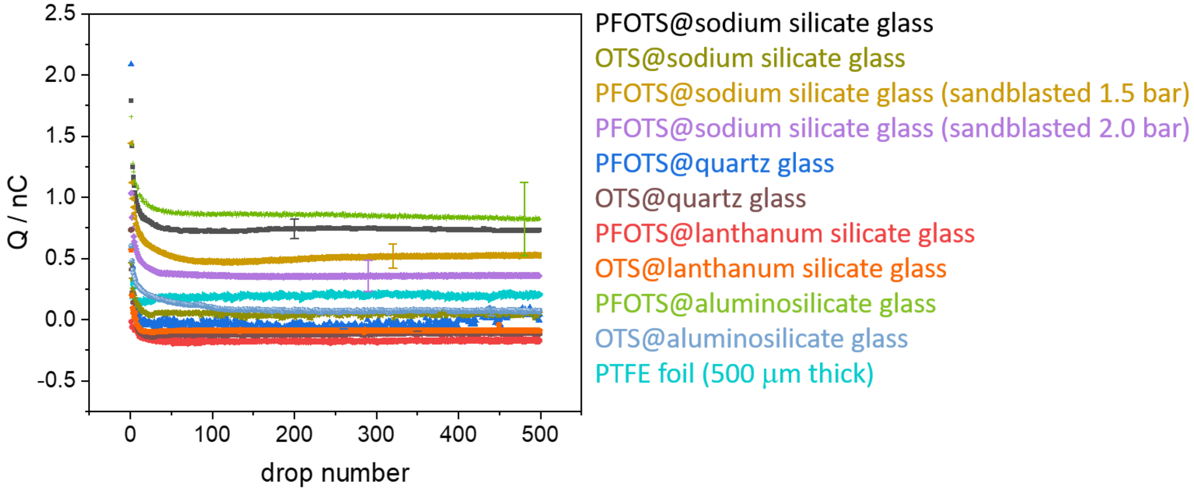

Figure S6. Averaged drop charge against the corresponding drop number of all measured samples. The calculation of the averaged drop charge is presented in Figure S5.

Table S7. Parameters of the Cauchy dispersion ($n(\lambda) = A + \frac{B}{\lambda^2} + \frac{C}{\lambda^4}$) and Urbach absorption tail ($k(\lambda) = k_1 \cdot e^{k_2(1240/\lambda - 1240/\lambda_b)}$) functions as determined from a fit of ellipsometry data.

Substrate or layer	A	B / nm^{-2}	C / nm^{-4}	k_1	k_2	λ_b / nm
quartz glass	1.443 ±4.357×10^{-5}	3710 ±183	1.37×10^{7} ±1.28×10^{6}	0.002 ±1.168×10^{-5}	0.725 ±0.062	250
sodium silicate glass	1.500 ±3.991×10^{-5}	5490 ±168	-1.36×10^{7} ±1.17×10^{6}	0.075 ±8.392×10^{-5}	0.608 ±0.001	250
lanthanum glass	1.769 ±3.022×10^{-4}	7730 ±1297	7.75×10^{8} ±9.28×10^{6}	0.132 ±9.581×10^{-5}	1.338 ±0.011	250
alumino-silicate glass	1.485 ±1.331×10^{-4}	6580 ±562	-2.59×10^{6} ±3.94×10^{6}	0.015 ±4.24×10^{-4}	1.786 ±0.069	250
OTS	1.419 ±3.592×10^{-4}	17300 ±2180	-1.43×10^{8} ±3.2×10^{7}			
PFTOS	1.300 ±6.784×10^{-4}	10830 ±3976	-4.69×10^{8} ±2.52×10^{7}			

AFM analysis of PFOTS@sodium silicate glass with a scan size 1μm x 1μm

The AFM measurements were done with the Bruker Dimension Icon with ScanAsyst device. The measurement were done with the Peak force mode in air. We used following cantilever: OLTESPA 70 kHz, 2 N/m; back side coating: reflective aluminum. The scan size was 1 μm x 1 μm.

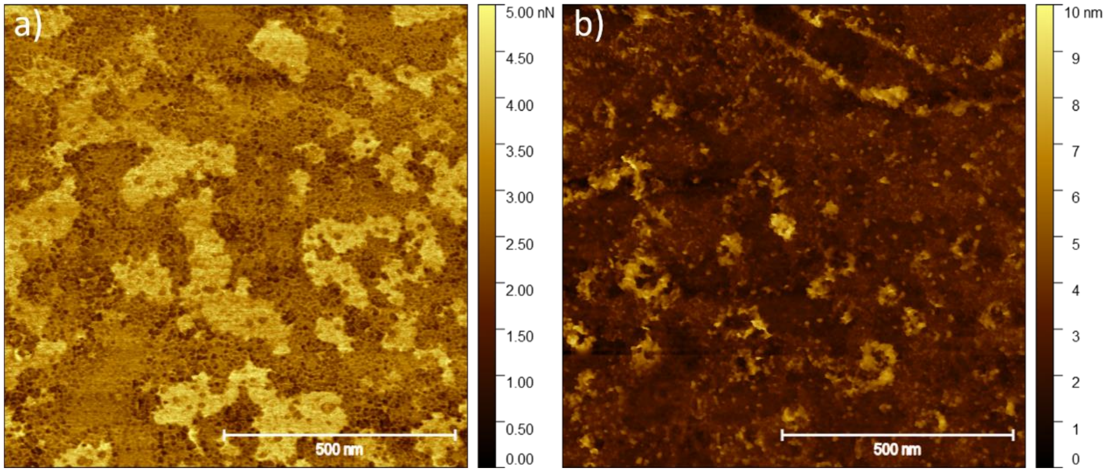

Figure S7. AFM images of the PFOTS coated sodium silicate surface. The AFM measurement were done in Peak force mode with a scan size of 1μm x 1 μm.

Model Structure and Length of an OTS and PFOTS Molecule on Glass

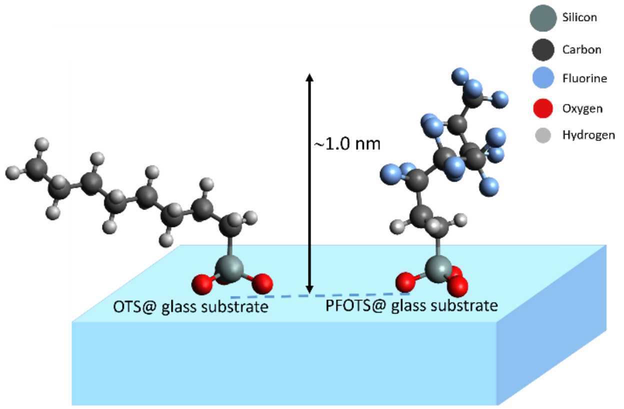

Figure S8. Model structure and length of OTS and PFOTS on glass. The molecule is bound to the three surface oxygen atoms. The calculation were done with the open source software Avogadro using UFF, a Universal Force Field. The calculation resulted in a length of 1 nm for an OTS molecule and a length of 1 nm for a PFOTS molecule bound to a glass surface.

XPS Surface Analysis

XPS was utilized to verify the formation of PFOTS and OTS layers on quartz glass, sodium silicate glass and lanthanum silicate glass samples Table S8. While the quartz glass samples show major contributions of silicon and oxygen, the surfaces of sodium silicate glass show additional contributions of sodium, potassium, calcium, and magnesium. The lanthanum silicate glass is characterized by additional amounts of lanthanum, boron, niobium, fluorine, and zinc. The formation of PFTOS and OTS silane layers is obvious from the decrease of the element content for the bare glass materials. In parallel, new signals appear from the formation of the PFTOS and OTS layers. The formation of PFOTS layer is accompanied by an increasing amount of fluorine. The appearance of distinct CF_2 and CF_3 signals in the C 1s (Figure 3) and the concomitant rise of different carbon species (Table S8) further demonstrates the formation of a PFOTS layer. The formation of an OTS layer can be demonstrated through the increasing carbon content above the level observed for the bare substrates. Notably, all PFTOS and OTS layers lack Cl 2p signals at about 199.0 eV (Figure S7), arising from Si-Cl units (Table S8) with amounts of chlorine below the detection limits of the XPS measurements. This shows the complete conversion of the silane precursors to single or multilayer siloxane structures.

Table S8. Binding energy and amount in atom% of chemical moieties present on the surfaces of quartz, sodium silicate and lanthanum silicate glasses before and after modification with PFTOS and OTS.

Region / Peak of found element	Binding energy (eV)	Bare quartz glass	Bare Na-silicate glass	Bare La-silicate glass	Bare Al-silicate glass	PFOTS@ Quartz glass	PFOTS@ Na-silicate glass	PFTOS@ La-silicate glass	PFTOS@ Al-silicate glass	OTS@ Quartz glass	OTS@ Na-silicate glass	OTS@ La-silicate glass	OTS@ Al-silicate glass
Al2p	75.1±0.2	-*	0.4	0.3	6.0	-	0.8	-	4.7	-	0.3	-	5.2
Si2p	103.4±0.1	35.2	31.2	13.1	22.6	25.9	17.1	11.1	21.8	31.1	30.2	12.2	23.6
B 1s	191.8±0.1	-	-	0.5	1.7	-	-	-	-	-	-	0.8	-
Nb 3d	207.2±0.1	-	-	2.6	-	-	-	1.7	-	-	-	2.8	-
C1s OTS C-Si	284.2±0.1	-	-	-	-	-	-	-	-	0.8	0.6	0.6	1.1
C1s PFTOS C-Si, C-C	285	1.3	2.1	3.1	5.2	1.4	2.8	5.4	1.4	6.3	4.1	5	7.3
C1s C*-, C-O	286.0±0.2	-	-	-	-	1.2	2.2	2.7	0.9	0.8	0.8	1.3	0.8
C1s C(=O)-O	289.3±0.2	-	-	-	-	-	-	-		-	-	0.8	-
C1s CF_2	291.2±0.1	-	-	-	-	4.9	9.3	8.1	2.5	-	-	-	-
C1s CF_3	293.6±0.2	-	-	-	-	1.1	2.1	1.8	0.4	-	-	-	-
K 2p	294.1±0.1	-	0.1	-	2.0	-	-	-	1.4	-	-	-	0.9
Zr3p	333.6±0.3	-	-	0.6	-	-	-	0.4	-	-	-	0.7	-
Ca 2p	347.2±0.5	-	1	7.1	0.3	-	0.6	3.7	-	-	-	6.5	-
Ti 2p	459.0±0.1	-	-	2.9	-	-	-	1.7	-	-	-	2.8	-
O 1s	532.3±0.8	63.6	64.1	63.2	56.9	46.7	33	36.9	54.5	61	63	61.4	58.2
F 1s F^-	685.8±0.1	-	-	2.7	3.4	-	-	-	-	-	-	-	2.0
F 1s F-C	688.5±0.1	-	-	-	-	18.9	31.8	25.2	11.4	-	-	2.1	-
La 3d	836.0±0.1	-	-	1.9	-	-	-	0.5	-	-	-	1.4	-
Zn 2p	1022.0±0.1	-	-	2.0		-	-	0.8	-	-	-	1.5	-
Na 1s	1072.5±0.1	-	0.8	-	1.4	-	-	-	0.7	-	0.9	-	0.6
Mg 1s	1304.7±0.2	-	0.4	-	0.5	-	0.4	-	0.3	-	0.2	-	0.3
Total amount:		100%	100%	100%	100%	100%	100%	100%	100%	100%	100%	100%	100%

*Values lower than detection limit of the XPS measurements, i.e. <0.1 atom %.

**The PFOTS and OTS layers formed on various glasses lack Si-Cl moieties from unreacted precursors.

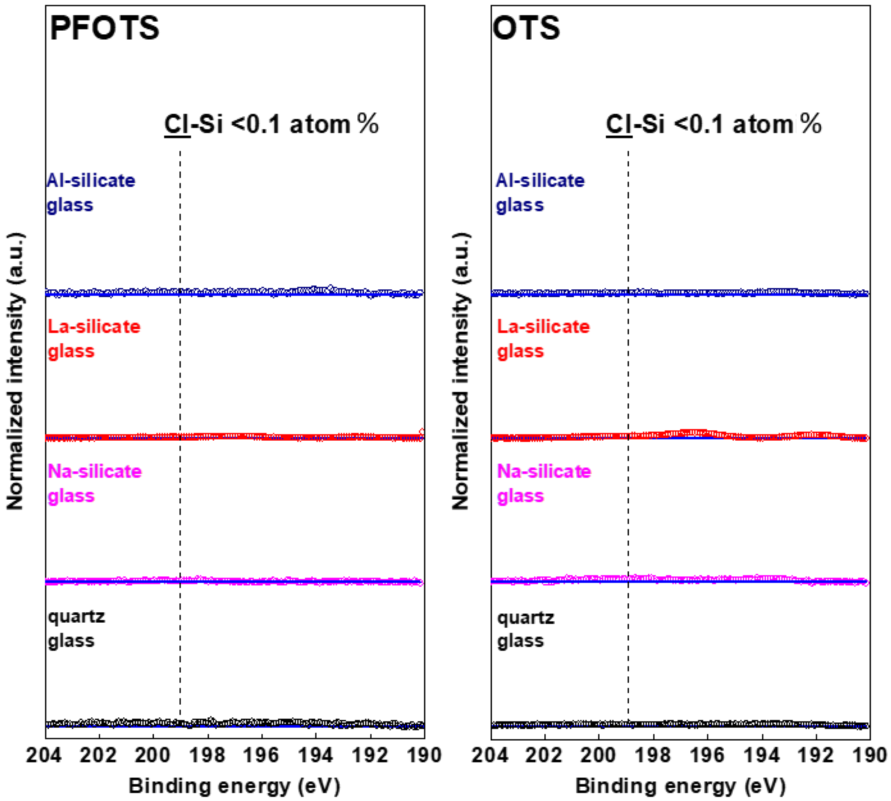

Figure S9. High-resolution XPS spectra in the Cl 2p region of PFOTS and OTS layers formed on quartz, sodium silicate and lanthanum silicate glass substrates. Measured spectra (open cycles) point to the absence of chlorine Cl-Si signals originating from precursor silane molecules thereby verifying the complete conversion of the silane precursors. The spectra are normalized to the maximum intensity observed in the C 1s spectra of the individual samples.

ζ Potential of Coated and Non-Coated Substrates

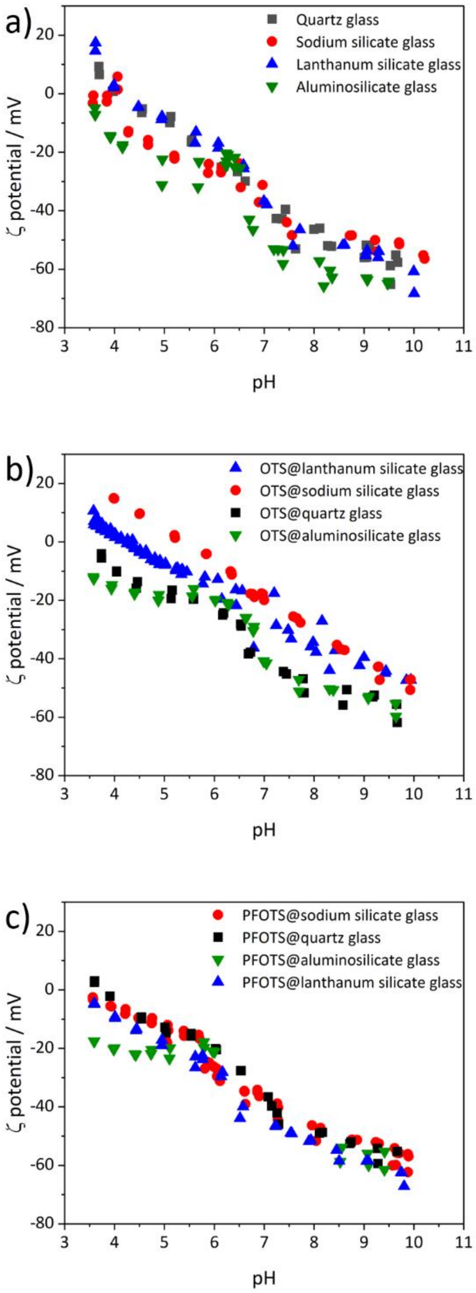

Figure S10. ζ potential of the coated and non-coated substrates in KCl solutions at constant conductivity of 10 mS·m^{-1} and different pH values to obtain the ζ potential titration curve. (a) ζ potential of the non-coated substrates. (b) ζ potential of the OTS coated substrates. (c) ζ potential of the PFOTS coated substrates. The measurement uncertainty was ±2 mV. Because of the small dimension of the measurement uncertainty and a better overview we did not plot the error in this figure.

Table S9. Weight (m), length (l), width (w), thickness (d) and calculated volume (V) of the glass substrates. The error of the volume was calculated with a Gaussian error propagation. The error of the respective measuring device was used as the error of the measured quantities. The density of the aluminosilicate glass was given by supplier (Schott AG).

Glass substrate	m / g	d / mm	w / mm	l / mm	V / cm^3
Quartz glass	4.57143±0.00001	1.08±0.01	25.44±0.01	76.22±0.01	2.209±0.002
Sodium silicate glass	4.71285±0.00001	1.02±0.01	25.77±0.01	75.76±0.01	1.991±0.002
Lanthanum silicate glass	10.34304±0.00001	1.51±0.01	25.07±0.01	76.00±0.01	2.899±0.002

Dielectric permittivity

The dielectric function $\varepsilon^*=C_p^*/C_0$ results from the measured complex sample capacity, C_p^*, where C_0 is provided by the sample geometry. This is the case of an ideal (i.e., perfectly homogeneous capacitor). A stray field contributes an additional external capacity to the ideal one (C_{edge}) as $C_{meas}^*= C_p^*+C_{edge}$. The latter contribution for a round plate capacitor is given by:

$$\frac{C_{edge}}{C_0} = \frac{2d}{\pi D}\left[ln\frac{8\pi D}{d} - 3 + (1+x)\ln(1+x) - xlnx\right] \quad (1)$$

Here, D is the electrode diameter, $x=t/D$ with t being the electrode thickness, and d is the sample thickness. The maximum error without the edge correction is given as

$$\frac{\Delta\varepsilon\prime}{\varepsilon\prime} = \frac{C_{edge}}{C_p'} = \frac{C_{edge}}{C_0\varepsilon'} \quad (2)$$

Employing typical values (t=1 mm, D=20 mm, d=1 mm and ε'=4) and Eqs 1 & 2 results to an uncertainty of 3.7%.

There are two ways to reduce this uncertainty in the permittivity values. One is to use a sample with guard ring. The second, employed by the Novocontrol set-up and used here, is to take into account in the evaluation of the capacity the edge correction as described above. With this feature enabled in the software, the overall uncertainty in the dielectric permittivity values is estimated to be about 1%. We measured every sample five times. For the result and discussion part we used the average values of this five measurements. The measurement uncertainty was calculated via standard deviation.

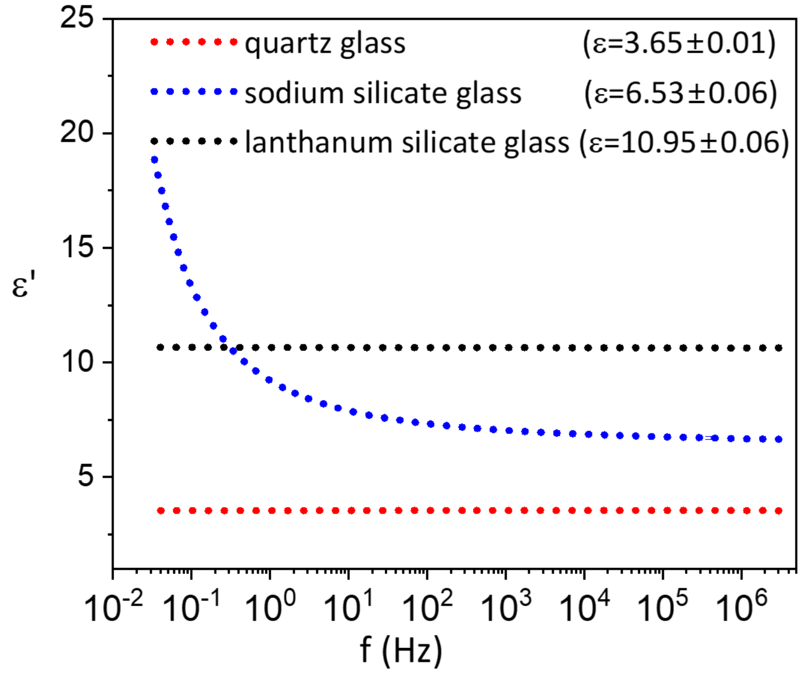

Figure S11. Dielectric spectra with corresponding permittivities ε of the quartz, sodium silicate and lanthanum silicate glass.
